# Machine Learning Models for Predicting Gynecological Cancers: Advances, Challenges, and Future Directions

**DOI:** 10.3390/cancers17172799

**Published:** 2025-08-27

**Authors:** Pankaj Garg, Madhu Krishna, Prakash Kulkarni, David Horne, Ravi Salgia, Sharad S. Singhal

**Affiliations:** 1 Department of Chemistry, GLA University, NH-19, Mathura-Delhi Road, Mathura 281406, Uttar Pradesh, India; 2 Department of Medical Oncology and Therapeutic Research, Beckman Research Institute of City of Hope, 1500 E Duarte Road, Duarte, CA 91010, USA; 3 Department of Molecular Medicine, Beckman Research Institute of City of Hope, 1500 E Duarte Road, Duarte, CA 91010, USA

**Keywords:** gynecological cancers, machine learning, early cancer detection, artificial intelligence in oncology, personalized medicine, multi-omics integration

## Abstract

Gynecological cancer, especially breast, cervical, and ovarian cancer, are significant health issues affecting women worldwide. When screened they are mostly detected at later stages because of non-specific signs and symptoms as well as the unavailability of reliable screening methods. The improvement of early oncologic prediction methods is therefore needed to work out the survival rates, guide individualized treatment, and relieve healthcare pressures. Outcome forecasting and clinical detection are rapidly changing with the use of machine learning (ML), one of the promising technologies used to analyze complex biomedical data. Artificial intelligence (AI)-based ML models are capable of determining low-level trends and making accurate predictions of disease risk and outcomes, because they can combine different datasets (clinical records, genomics, proteomics, medical imaging) and learn to identify subtle patterns. Standard algorithms, including support vector machines, random forests, and deep learning (DL) models, such as convolutional neural networks, have demonstrated high potential in identifying the type of cancer, monitoring disease progression, and designing treatment patterns. This manuscript reviews the recent developments in the use of ML models to advance oncologic prediction tasks in gynecologic oncology. It reports on critical domains, like screening, risk classification, and survival modeling, as well as comments on difficulties, like data inconsistency, inability of interpretation of models, and issues of clinical interpretation. New developments, such as explainable AI, federated learning (FL), and multi-omics fusion, are discussed to develop these models and to make them applicable in practice because of their reliability. Conclusively, this article emphasizes the transformative role of ML in precision oncology to deliver improved, patient-centered outcomes to women who are victims of gynecological cancers.

## Simple Summary

This review explores how machine learning, an advanced computer-based method, is changing the way healthcare professionals detect and treat women’s cancers like breast, cervical, and ovarian cancer. These smart tools can study medical images, lab results, and patient history to help perceive cancer early, support outcome forecasting, predict how cancer might grow, and choose the best treatment. This article explains how these systems work, shares real-life examples, and highlights both the benefits and challenges of machine learning. It ends by showing how, with better data and safer systems, machine learning could become a powerful partner in providing faster, more accurate, and personalized cancer care.

## 1. Introduction

Gynecological cancer, including breast cancer (BC), ovarian cancer (OC), and cervical cancer, is a major health burden for women worldwide. BC is the most common gynecological cancer and remains a leading cause of cancer mortality. OC, though less common, is typically detected at advanced stages due to its silent progression, resulting in poor survival [1]. Cervical cancer is largely preventable with HPV vaccination and frequent screening. Nevertheless, access remains limited in many low- and middle-income countries. Collectively, these cancers pose substantial physical, emotional, social, and economic burdens. Late detection remains a key challenge. Symptoms are often nonspecific and emerge late, reducing treatment effectiveness and survival rates. For gynecologic oncologists, late detection remains a challenge, making accurate and predictive diagnostic tools essential for improving survival and fertility-sparing options [2].

Conventional diagnostic methods often lack sensitivity, particularly for early or aggressive subtypes. Patient responses to standard treatments vary, underscoring the importance of personalized medicine [3]. Therefore, there is an urgent need for tools that enable early prognosis, individualized risk assessment, and treatment selection based on a patient’s unique clinical and biological profile. This is the area where predictive technologies such as machine learning (ML) are proving transformative.

ML, a subfield of artificial intelligence (AI), offers powerful capabilities for analyzing complex medical data. Unlike traditional statistical approaches, ML can identify patterns in large, high-dimensional datasets such as clinical records, genomic profiles, imaging, and biosignals. ML has been applied across the cancer care continuum, from disease prognosis and risk assessment to treatment planning and survival prediction [4]. ML models have shown great potential in gynecologic oncology, classifying tumor types, automating Pap smear and mammogram interpretation, predicting metastasis or recurrence, and discovering novel biomarkers from genomic data. Such applications support more proactive, accurate, and evidence-based cancer care [5].

Despite several reviews on AI and oncology, there remains a distinct gap in focusing specifically on gynecological cancers, which present unique challenges such as late detection, fertility-sparing treatment needs, and disparities in access to care. Prior works have either generalized across all cancers or emphasized technical algorithms without sufficient clinical context. This review addresses that gap by (i) providing a comparative overview of both traditional ML (e.g., logistic regression, support vector machines (SVMs), random forests) and deep learning (DL) models (convolutional neural networks (CNNs), recurrent neural networks (RNNs), transformers) in gynecologic oncology, and (ii) integrating insights from a gynecologic oncology perspective often missing in previous reviews. Furthermore, recent advancements in spectral and hyperspectral DL models for early disease detection, such as those by Tsai et al. (2025) and Huang et al. (2025), illustrate innovations that could be translated into gynecological cancer detection, further underscoring the novelty and timeliness of this review [6,7].

This review summarizes ML applications for the early diagnosis and management of BC, OC, and cervical cancer. It aims to bridge the gap between biomedical and computational fields, presenting current advancements, real-world applications, and limitations. We examine algorithm types, dataset characteristics, and cancer-specific challenges [8]. We also highlight issues such as dataset bias, lack of interpretability, and barriers to clinical integration. Finally, we outline future prospects for ML in gynecology, emphasizing its potential to advance personalized care, promote health equity, and enable next-generation diagnostic tools. Our goal is to guide future research and foster collaboration in combating gynecologic cancers [9].

### Search Strategy and Scope

Although this review is narrative in nature, we applied a defined selection process. We included peer-reviewed articles published between 2015 and 2025 that specifically addressed ML in gynecological cancers. Studies were considered if they employed ML or DL for oncologic prediction tasks, clinical detection, outcome forecasting, or treatment stratification, with preference given to works integrating clinical, imaging, or biomarker data. We excluded purely methodological computer science papers without clinical relevance. Both classical ML algorithms (e.g., logistic regression, SVMs, random forests) and advanced DL approaches (e.g., CNNs, RNNs, transformers) were included to ensure balanced coverage and minimize selection bias.

## 2. Overview of Gynecological Cancers

### 2.1. Breast Cancer (BC)

BC is the most frequently identified cancer in women all over the world and it is on the frontline as a major issue of concern to population health. Heterogeneity defined by hormone receptor and HER2 status drives clinical risk stratification and treatment choices. The treatment decisions for BC are based on these molecular subtypes, and they have influence on the outcome forecasting [10]. Mammography enables earlier monitoring; however, underserved groups often present late, thus reducing survival rates. Although advancements in BC screening and targeted therapies have significantly improved survival, ML can help integrate imaging, genomic, and clinical data to improve oncologic prediction tasks, treatment response, and survival [11].

### 2.2. Ovarian Cancer (OC)

OC, commonly recognized as a ‘silent killer’, is usually diagnosed late due to vague symptoms and often presents at advanced stage. Epithelial OC (EOC) is the most frequent type, comprising more than 90% of the OC cases. Despite lower incidence, OC accounts for a disproportionate share of deaths due to lack of initial diagnosis [12]. Although some gene mutations, including BRCA1/2, have been associated with increased risk, screening remains inadequate. ML is being explored to analyze genomic/proteomic/imaging data for subtle early markers, enhancing risk stratification and supporting earlier interventions [13].

### 2.3. Cervical Cancer

Cervical cancer remains highly preventable with HPV vaccination and screening, yet it still ranks among the leading causes of female cancer mortality, especially in low-resource regions. It most often arises as squamous cell carcinoma or adenocarcinoma, which are strongly linked to chronic HPV infection. HPV-DNA testing and Pap smear screening have substantially reduced incidence in high-income countries, but benefits remain limited in low-resource settings [14]. The problem is how to extend these benefits to low-resource settings where screening is often rare or even absent. In addition, Pap smear and colposcopy images can be interpreted subjectively, making them dependent on the expertise of the clinician. ML is being used to automate Pap smear/colposcopy image analysis and integrate biomarkers for objective, accessible screening in under-resourced settings [15]. These three, BC, OC, and cervical cancers, illustrate where ML could bridge gaps in early detection, outcome forecasting, and personalized treatment strategies.

## 3. ML Methods in the Prediction of Cancers in Gynecology

Over the past few years, ML has become integral to oncology, enabling risk prediction, diagnostics, and individualized treatment planning. The main approaches are supervised, unsupervised, and DL, with hybrid extensions. Each approach has its reasoning, advantages, and possible areas of usage, in particular, clinical and biomedical studies. Table 1 summarizes key categories of ML approaches with examples in gynecologic oncology [16,17].

### 3.1. Supervised Learning: Example-Based Learning

Supervised learning is the most intuitive ML approach, where algorithms learn from labeled datasets. For instance, a dataset of patient records includes age, tumor size, hormone receptor status, and whether cancer is present [18]. The model is trained to link clinical features with outcomes, enabling prediction in new patients. When trained, the model can forecast the likelihood of new patients having cancer depending on their clinical data. From a gynecologic oncologist’s perspective, supervised learning enables more personalized patient management. For example, using logistic regression on preoperative CA-125 levels and imaging data can help identify high-risk OC patients who may benefit from neoadjuvant chemotherapy before surgery. The main supervised algorithms include decision trees, support vector machines, and random forests [19].

#### 3.1.1. Decision Trees: Clear and Easy Tools for Gynecological Cancer Prediction

Decision trees are simple ML models that mimic clinical reasoning by asking sequential yes/no questions (e.g., tumor > 2 cm, HPV-positive) until reaching a diagnosis [20]. Their transparency makes them attractive for oncology, where interpretability is essential. In BC, they classify mammographic features such as tissue density, shape, and margins [21]. In cervical cancer, they integrate Pap smear results, history, and HPV genotype for risk assessment. For OC, they help differentiate benign vs. malignant cysts using imaging and clinical findings [22,23]. One study achieved > 80% accuracy in classifying cervical cancer risk from clinical and demographic data. Such models have their highest accuracy when applied to well-labeled and high-quality datasets that include diagnostic images, genomic and proteomic profiles, clinical records and pathological reports, and histological findings. Their ability to explain decisions bridges AI outputs with physician reasoning, which is exceptionally advantageous when there is a gap between AI and clinical reasoning in circumstances where physician review is required [23].

#### 3.1.2. Support Vector Machines (SVMs): Drawing Smart Boundaries for Cancer Prediction

SVMs are high-precision algorithms that classify data by drawing an optimal boundary (hyperplane). Using kernel functions, they handle complex or non-linear data, enabling the detection of subtle clinical patterns [24]. The role of SVMs has been proven valuable in the context of gynecological cancer. In BC, they analyze mammograms and ultrasounds by assessing tumor size, margins, and tissue texture. SVMs are also used on other data types, such as the expression values of molecules (HER2, ER, PR), to aid in the subclassification of cancer and assist in treatment strategies [25]. For example, SVMs have been used to identify small lesions of malignancy in mammograms that were not detected in early screening, thus representing the worth of SVMs in the early forecasting of disease [26].

In cervical cancer, SVMs automate Pap smear analysis, improving accuracy in distinguishing normal from abnormal cells [27]. They also estimate CIN2+ risk using patient history and high-risk HPV genotypes. These tools have the potential to enhance screening and facilitate the practice of early intervention, especially in low-resource situations. For OC, SVMs analyze CA-125, HE4, and microRNAs to distinguish early cancer from benign cysts [28]. Combining proteomic or metabolomic data improves diagnostic sensitivity and reduces false positives, avoiding unnecessary surgery [29]. SVMs are also very useful in the medical field, as they even perform well using small or degree-limited data, so they can be used before a product is fully researched. They also cope with high-dimensional data easily, which is typical of genomic and biomarker studies [30]. Moreover, they resist overfitting, yielding reliable predictions across patient groups. These advantages make SVMs one of the most indispensable tools in the ML arsenal for cancer risk stratification and survival outcome.

#### 3.1.3. Random Forests: Collective Intelligence for the Prediction of Cancer

Random forest (RF) refers to a kind of collective ML that computes an ensemble of decision trees, each grown on subsets of the data, performing a sort of vote to produce a more precise and final prediction [31]. This collaborative decision-making model minimizes overfitting and improves the generalizability of the model, making it very efficient in processing complex biomedical data. In gynecologic oncology, random forests are used to predict BC recurrence and survival using genetic and histological features [32]. They can also integrate large-scale clinical data to stratify patients into risk groups and guide individualized treatment.

In cervical cancer, random forests automate Pap smear evaluation with high sensitivity, improving diagnostic consistency and reducing cytologist workload [33]. For OC, they combine ultrasound features and blood biomarkers to assess malignancy risk. Random forest models are powerful because they can process a variety of information types, including imaging, genomics, or clinical parameters, and these models can detect very nuanced interactivity between variables that might not be evident in standard approaches [34]. A notable example from The Cancer Genome Atlas (TCGA) showed random forests classifying BC subtypes from gene expression, supporting molecular diagnostics and precision treatment [35]. Their flexibility, stability, and ability to rank variable importance make them valuable for biomarker discovery and for providing transparency in clinical decision-making [36].

### 3.2. Unsupervised Learning: Bringing to Light the Hidden Structure of Cancer Data

Unsupervised learning works without labeled outcomes, exploring data to uncover hidden patterns or groups [37]. For example, unsupervised learning can cluster patient records based on shared traits without prior knowledge of disease diagnosis and risk assessment. In gynecologic oncology, it can reveal subtle molecular variations, such as new ovarian or endometrial cancer subtypes, from genomic/proteomic data, offering insights into tumor biology and treatment targets [38]. Key methods include clustering (e.g., grouping by gene expression to define subtypes) and hierarchical clustering, which maps patient relationships in tree-like structures [39]. Clinically, unsupervised learning can identify patient subgroups with distinct responses. For instance, clustering gene expression profiles from endometrial cancer biopsies can reveal molecular subtypes that guide targeted therapy selection.

Principal component analysis (PCA) is another useful tool that simplifies the representation of data when dimensions are large, keeping the most significant trends and making visualization and interpretation of higher-dimension data simpler than in genomics or proteomics [40]. These methods help discover biomarkers, classify tumor subtypes, and clarify genetic diversity, especially in hard-to-detect ovarian and endometrial cancers. Ultimately, unsupervised learning supports early recognition, individualized treatment, and deeper biological insights by exposing hidden structures in complex data [41].

### 3.3. Deep Learning (DL): Emulate the Brain to Crack the Code of Cancer Complexity

DL, inspired by the human brain, uses multi-layered neural networks to analyze unstructured and complex data, such as medical images, histopathology slides, and genomic sequences [42]. Unlike traditional ML, DL automatically extracts meaningful features from raw data, reducing preprocessing needs and excelling at tasks like image classification and pattern recognition [43].

DL is being utilized in research on gynecological cancers, both for enhancing diagnosis and prediction outcomes. DL is especially utilized in image-based tasks, with convolutional neural networks (CNNs) representing one of the most-used architectures. These models have demonstrated great success in detecting tumors in mammograms, detecting cervical cell images using Pap smears, and detecting early stages of lesions using visual inspection photographs [44]. For a gynecologic oncologist, DL models such as CNNs can automate the detection of subtle abnormalities in Pap smear or colposcopy images, allowing earlier referral for diagnostic biopsy and reducing the risk of missed high-grade lesions. A variant of DL, recurrent neural networks (RNNs), analyze sequential data such as patient vitals or gene expression across disease stages. DL also assists in digital histopathology, detecting cancerous regions overlooked by the human eye. In addition, DL is increasingly used to integrate different types of biological data, such as genomics, proteomics, and even metabolomics, to better predict disease outcomes and design individualized therapies [45]. Such models enhance diagnostic accuracy, reduce human error, and support earlier intervention as they continue to evolve in gynecologic oncology. An overview of various ML algorithms employed across gynecological cancers, highlighting their applications and clinical relevance, is presented in Table 2.

## 4. The Practice of ML in Prediction of Gynecological Cancers

**Figure 1:**
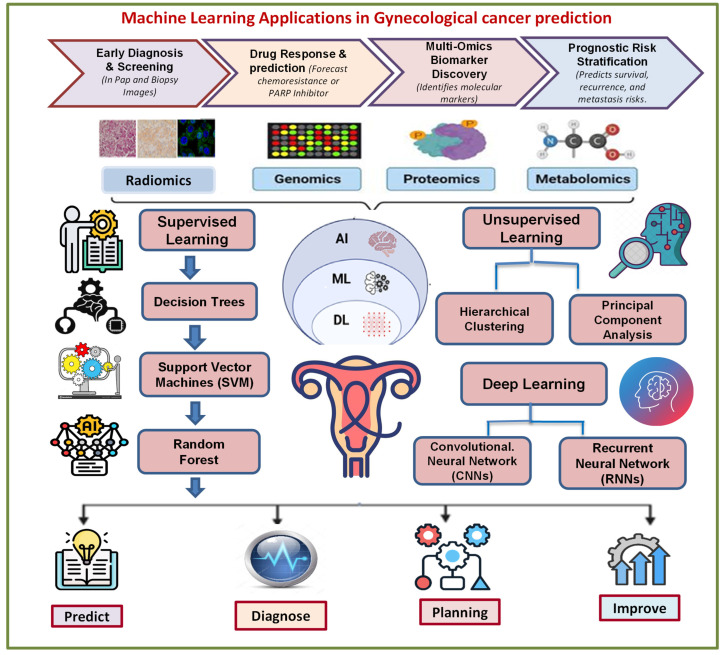
Conceptual workflow of ML approaches in gynecological cancer prediction. This figure presents a streamlined overview of how ML methodologies operate in gynecological cancer care, from data input to clinical output. It highlights the integration of diverse datasets including clinical records, imaging, genomics, and proteomics into different ML methodologies such as supervised (Section 3.1), unsupervised (Section 3.2), and DL (Section 3.3). The resulting predictions, early diagnosis, risk stratification, and treatment guidance, are fed into clinical decision support systems (CDSS) and electronic health records (EHRs) for real-time application. This workflow underlines the core premise of the review, that ML can transform fragmented, complex medical data into actionable insights for personalized and efficient gynecologic cancer care.

ML is revolutionizing gynecological oncology by enabling earlier and more personalized detection and treatment [57]. These systems process large clinical, imaging, and molecular datasets, revealing patterns beyond traditional methods. The sections below discuss the applicable use of ML in forecasting each of these cancers, including the nature of the data, the algorithm being employed, and their clinical value [3,50,58]. The stepwise role of ML approaches in gynecological cancer extrapolation, from data input to integration into clinical tools, is illustrated in Figure 1**.**

### 4.1. BC: Early Diagnosis and Personalization

ML is accelerating BC management by supporting diagnosis, risk assessment, and treatment planning [59].

#### 4.1.1. ML-Based Imaging-Based Diagnosis

Mammography, ultrasound, MRI, and other methods of medical imaging have traditionally been the mainstays of BC screening. CNNs achieve > 90% accuracy in distinguishing benign from malignant lesions [47]. Combining imaging with genomic and histopathological data further strengthens diagnostic reliability [48,60].

Clinical relevance: In clinical breast oncology practice, such CNN-based imaging models could assist radiologists and oncologists in reducing false negatives in mammography and ensuring timely biopsy referrals, ultimately improving disease prognosis and surgical planning.

#### 4.1.2. Profiling by Genomic and Transcriptomic Data

BC is genetically heterogeneous, and elucidation of the molecular nature of this cancer is imperative for precision treatment [61]. ML algorithms (RF, SVM, deep networks) trained on TCGA-BRCA and METABRIC datasets stratify tumor subtypes such as ER+, HER2+, and triple-negative, informing therapy and prognosis [46,62,63].

Clinical relevance: For breast oncologists, genomic profiling guided by ML not only supports therapy decisions (e.g., HER2-targeted treatment) but also helps identify patients suitable for fertility-sparing strategies or closer follow-up in high-risk cases.

#### 4.1.3. Risk Predictive Assessment

ML is used to predict BC risk based on family history of cancer, BRCA1 or BRCA2 mutation status, hormonal makeup, and lifestyle to produce individualized risk scores [64]. Dynamic models such as BOADICEA and Tyrer–Cuzick achieve higher predictive accuracy and support early screening and counseling [65].

Clinical relevance: In a clinical workflow, these predictive models help oncologists stratify women into high-risk categories, ensuring genetic counseling and surveillance are prioritized for those most likely to benefit.

### 4.2. Cervical Cancer: ML Efficacy to Improve Prevention and Detection

Most cervical cancers arise from persistent high-risk HPV infection; ML enhances initial finding, prevention, and risk estimation [64,65,66]. ML is already having a lifesaving, game-changing impact in this domain, as it is empowering a more specific, efficient, and personalized methodology of screening and prevention.

#### 4.2.1. HPV and Screening Statistical Analysis

ML algorithms (decision trees, logistic regression, XGBoost) can predict CIN2+ progression with >85% sensitivity [56,67,68].

Clinical relevance: Clinically, this enables gynecologic oncologists to tailor screening intervals, identify women at highest risk for CIN2+ progression, and initiate earlier preventive interventions.

#### 4.2.2. Pap Smear Image Interpretation

DL models such as CNNs (U-Net, VGGNet) automate cytology image analysis, reducing human error and supporting initial diagnosis, especially in low-resource settings [49,69].

Clinical relevance: In a gynecologic oncology setting, an SVM-based cytology classifier can rapidly flag suspicious Pap smears for expedited colposcopy, reducing diagnostic delays and enabling earlier intervention.

#### 4.2.3. Risk Stratification via Clinical and Behavioral Data

ML models now integrate behavioral and social variables (e.g., smoking, contraceptive use, socioeconomic status) with biological markers, creating comprehensive risk profiles [70,71].

Clinical relevance: From a gynecologic oncology standpoint, these integrated risk models provide decision support for counseling patients, optimizing screening schedules, and guiding HPV vaccination outreach strategies.

### 4.3. OC: Early Detection and Prognosis

OC is one of the most difficult cases of gynecological malignancy to recognize at early stages because of non-specific and mild symptoms. As a result, OC is often diagnosed at late stages, leading to poor prognosis. ML offers new approaches through biomarkers, imaging, and prognostic modeling [72].

#### 4.3.1. Exploring Biomarkers for Early Recognition

Least absolute shrinkage and selection operator (LASSO), SVMs, and RF models analyze microRNA, proteomic, and metabolomic data to identify early biomarkers. Combining CA-125 with microRNA improved the specificity of early OC detection to 93% [51,52,53].

Clinical relevance: For gynecologic oncologists, integrating CA-125 with novel molecular biomarkers through ML allows for earlier triaging of suspected OC cases and facilitates decisions on neoadjuvant chemotherapy versus upfront surgery.

#### 4.3.2. Sophisticated Imaging and Radiomics Usages

Radiomics extracts quantitative features from MRI/CT to distinguish benign from malignant tumors; radiogenomics links imaging with BRCA1/2 status [73].

Clinical relevance: For gynecologic oncologists, RF-derived staging from MRI radiomics allows for preoperative surgical planning, including decisions on lymphadenectomy and fertility-sparing options. This combination of both radiologic and genomic data provides an additional level to personalized imaging, which may lead to real-time therapy choice and risk stratification [74].

#### 4.3.3. Prognostic Modeling and Survival Prediction

Random survival forests and Cox ML models predict recurrence-free survival and platinum resistance using tumor stage, histology, and molecular markers [54,75].

Clinical relevance: In routine gynecologic oncology practice, such prognostic models enable risk-adapted follow-up, inform the choice of second-line therapies, and support patient-centered discussions about expected outcomes. Table 3 summarizes the clinical applications of ML techniques in different gynecological cancers, detailing the type of data utilized, algorithms implemented, and corresponding clinical impact.

## 5. Key Challenges and Limitations in ML Adoption in Oncology

Although ML has great promise in gynecologic oncology, its clinical integration remains limited. While it can aid in earlier diagnosis, risk stratification, and individualized therapy, several systemic barriers slow real-world adoption. These include data quality, algorithm reliability, validation, infrastructure, regulatory oversight, ethics, and limited clinical training [77,78]. Table 4 provides an overview of ML approaches, their advantages, and readiness for clinical use.

### 5.1. Data-Related Challenges

The quality and inconsistency of data are among the most critical barriers. In gynecologic oncology, incomplete records, inconsistent labeling, and class imbalance (e.g., more early-stage than rare, advanced cases) reduce model reliability and generalizability [79,80]. Most datasets come from high-income regions, limiting global applicability, while strict laws (HIPAA, GDPR) further hinder collaboration, especially in low- and middle-income countries.

Many ML studies in gynecological oncology face limitations beyond general data inconsistency. Small sample sizes, especially in rare subtypes such as clear-cell OC, restrict model robustness. Severe class imbalance between early- and late-stage cases often skews predictions toward advanced disease, reducing early-detection sensitivity. Domain shifts across institutions, stemming from differences in imaging equipment, staining protocols, or population demographics, further compromise model generalizability. Addressing these issues will require federated, multi-center datasets and harmonization strategies to enable clinically reliable models.

### 5.2. Model-Related Challenges

In the field of oncology, challenges associated with modeling restrain the clinical implementation of ML. A major challenge is interpretability; many ML tools act as “black boxes,” producing outputs without transparent reasoning [76]. This limits clinical trust, especially in gynecologic oncology decision-making. Overfitting is another concern; for instance, a Pap smear model trained in one lab may fail elsewhere due to staining variability [81]. Furthermore, most ML tools lack prospective validation, being tested only retrospectively, which weakens confidence in real-world oncology use [82].

### 5.3. Clinical Integration and Infrastructure Barriers

Workflow disruption is another barrier. Even accurate models may fail if not well integrated into electronic health records (EHRs) or if they increase clinician workload [83]. Regulatory uncertainty also delays adoption, as agencies like the FDA and EMA lack clear approval pathways for AI-based oncology tools [84].

### 5.4. Ethical, Legal and Social Considerations

There are some noteworthy ethical, legal, and social issues that come up in applying ML in gynecological oncology. Algorithmic bias may worsen inequities, especially for underrepresented groups [85]. In addition, a significant number of patients are unaware of what happens to their data, and they are not informed of the effects of ML on their diagnosis or treatment regimes. Privacy risks are high, as ML relies on sensitive genomic and imaging data. A lack of transparency around how ML influences diagnosis and treatment also threatens informed consent and patient trust [55].

### 5.5. Resource Constraints and Education Gaps

Resource constraints and inadequate professional training are some of the factors that hinder the development and implementation of ML in gynecological oncology [86]. Developing and maintaining ML models demands costly infrastructure and expertise, often unavailable in low-resource settings [86]. Clinicians also lack sufficient AI training, limiting their ability to interpret and trust ML tools. Slow integration of digital health into medical education compounds this gap [87].

### 5.6. Responsible and Equitable Integration

ML represents an extraordinary opportunity in revolutionizing the work of gynecological oncology, but its effective on-the-ground use depends substantially on sustaining various multifaceted challenges. Effective adoption will require diverse, representative datasets, interdisciplinary collaboration, regulatory clarity, and integration into clinical workflows. Investment in clinician AI education and digital literacy is equally vital. Only through these steps can ML become a reliable, equitable tool in gynecologic oncology for diagnosis, personalized treatment, and improved patient outcomes [88,89].

### 5.7. Benchmarking and Validation Limitations

Another limitation across existing studies is the inconsistency in reported performance metrics. While some use AUC and accuracy, others emphasize sensitivity, specificity, or F1-score, making cross-comparison difficult. Moreover, external validation using independent datasets or multi-center cohorts is rare, raising concerns about model generalizability. Without standardized benchmarking, the true comparative performance of ML and DL methods in gynecologic oncology remains unclear (Table 5).

## 6. Gynecological Cancer Care: Future Directions and Opportunities in ML

**Figure 2:**
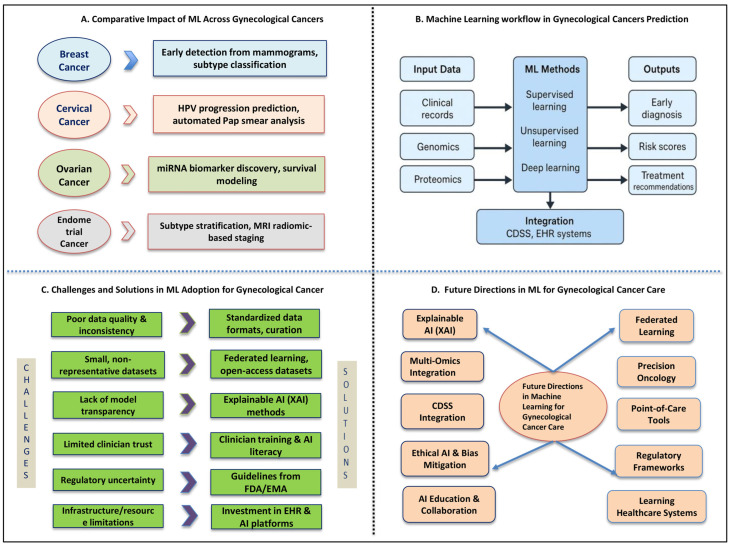
Multidimensional insights into the future of ML in gynecological oncology.

ML technologies are continually developing, and their integration into gynecological oncology holds enormous promise for transforming care, enabling earlier detection, guiding therapy decisions, and supporting equitable, personalized treatment delivery [90]. Even though important advances have been made, several emerging trends will define the next innovation phase in gynecologic oncology [91]. The key futuristic opportunities span explainability, federated data sharing, multi-omics, personalized medicine, workflow integration, and ethical AI, summarized in Figure 2.

This multi-panel figure illustrates a holistic view of how ML is transforming gynecological oncology. Panel A presents a comparative overview of ML applications across various gynecological cancers, including breast, cervical, ovarian, and endometrial cancer. It highlights differences in data sources (e.g., imaging, genomics, histopathology) and how ML supports tumor detection, subtype classification, recurrence prediction, and early screening, underscoring the versatility of ML models in diverse clinical contexts. Panel B visualizes the progressive ML workflow, emphasizing the cyclical process of learning from data, predicting outcomes, classifying cancer subtypes, and continuously improving accuracy. This schematic captures the core engine behind ML performance and its adaptability across different stages of cancer care. Panel C outlines the key challenges hindering widespread ML adoption in oncology, such as data quality issues, algorithmic bias, limited interpretability, and lack of clinical validation. It also proposes practical solutions, including cross-disciplinary collaboration, regulatory clarity, and clinician education, to bridge the gap between academic promise and clinical reality. Panel D looks ahead to future opportunities in the field. It highlights emerging trends such as explainable AI, federated learning, integration of multi-omics and real-world data, and personalized decision-supporting systems. These innovations signal the next frontier of ML-driven cancer care, aimed at delivering more equitable, transparent, and individualized patient outcomes.

### 6.1. On the Way to Explainable and Trustworthy AI

Increasing model transparency and interpretability remains central for clinical adoption, since oncologists must understand why a model recommends a given diagnosis or therapy. Explainable AI (XAI) aims to provide clinically meaningful explanations, such as highlighting mammogram features that triggered a high-risk classification. Methods such as Shapley additive explanations (SHAP) and local interpretable model-agnostic explanations (LIME) are growing in popularity, as they allow black-box models to become more interpretable [92]. These tools can help close the trust gap and support confident decision-making in gynecologic oncology. While interpretability tools such as SHAP and LIME are promising, their clinical validation remains limited. Few gynecologic oncology studies have tested whether these explanations truly enhance clinician trust, diagnostic accuracy, or decision-making efficiency. Evidence from early pilot studies in oncology suggests interpretability may improve physician confidence in model outputs, but large-scale trials are still lacking. Thus, explainable AI remains an important but largely theoretical innovation in this field, and bridging this gap will be essential for clinical adoption.

### 6.2. Learning Federated and Safe Data Sharing

To build strong ML models, access to diverse, representative data is essential, but data sharing is limited by privacy and IP restrictions. The solution provided by federated learning (FL) allows for model training across institutions without exchanging raw patient data [93]. It protects patient privacy while enabling global collaboration. Due to its unique benefits, FL can generate models representative of diverse populations, improving generalizability in gynecologic oncology [94].

### 6.3. Multi-Omics and Real-World Data Integration

Integration of multi-omics with real-world clinical data is another frontier. Integration of multi-omics, that is, genomics, proteomics, transcriptomics, and metabolomics can yield a deeper view of tumor biology [95]. Combined with real-world data, including lifestyle, environmental, EHR, and wearable sensor data, ML may uncover new predictors of cancer risk, progression, and therapy response, enhancing premature recognition and prognostic modeling in gynecologic cancers [96].

### 6.4. Personalized and Precision Oncology

ML is central to advancing precision oncology, tailoring interventions to each patient’s molecular and clinical profile [97]. These interventions include predictive models that identify patients most likely to benefit from hormone therapy, chemotherapy, or immunotherapy. They also can forecast recurrence risk, support fertility-sparing choices in younger women, and personalize surveillance schedules. In gynecologic oncology, such personalization improves outcomes while avoiding unnecessary interventions [98].

### 6.5. The Clinical Decision Support Systems (CDSS)

With the advancement in ML, real-time CDSS will become more widely used. These systems can deliver actionable insights at the point of care, flagging high-risk imaging, pathology, or EHR findings during consultations or surgery [99]. Further, user-friendly tools (e.g., dashboards, mobile apps, or voice-assisted systems) are needed to streamline workflows without adding clinician burden [100].

### 6.6. Point of Care and Resource-Limited Uses

Most low-resource environments lack adequate specialists and equipment for gynecologic cancer screening. ML provides scalable solutions that democratize access to healthcare [101]. For example, ML-enabled smartphone cervical imaging or AI-assisted HPV self-sampling can help provide underserved communities with life-saving early detection tools. Besides aiding in decreased disparities in health care, these developments may lead to early intervention, which could enhance survival rates in areas where late-stage diagnosis is still prevalent [102].

### 6.7. Ethical AI and Biases Reduction

With the increasing integration of ML in cancer care, ensuring ethical, bias-free AI is essential. Algorithm bias remains a major issue, as models trained on non-representative data may worsen disparities [103]. Future development must prioritize diverse datasets, bias-detection tools, and validation across populations to ensure equitable benefits [104].

### 6.8. Policy Development and Regulation Frameworks

Robust regulatory frameworks are needed to ensure ML tools are safe, effective, and ethical. As the field matures, models may undergo trials, post-market surveillance, and continuous monitoring. Well-documented guidelines provided by agencies like FDA and EMA, along with universal benchmarks, will build clinician confidence in AI adoption [105].

### 6.9. Cross-Disciplinary Collaboration and Education

Maximizing ML’s role requires education and cross-disciplinary collaboration. Clinicians need AI literacy, while data scientists must understand clinical oncology. Medical students, residents, and other healthcare practitioners, as well as partnerships among oncologists, bioinformaticians, ethicists, and engineers should drive training programs to close this knowledge gap [106].

### 6.10. On the Way to Learning Healthcare System

The most transformative vision for ML is a learning healthcare system, one continuously improving as new clinical data is added. Every patient encounter, imaging study, or lab test contributes to iterative model refinement, enabling real-time improvement of care. Such a feedback loop creates a dynamic system that grows more precise, efficient, and personalized with every cycle [107].

The potential of ML in gynecologic oncology is both promising and transformative. Equitable early diagnosis and personalized treatment are becoming attainable, but success depends on responsible innovation, collaboration, regulation, and commitment to transparency and patient-centered care. If achieved, ML could become a foundational pillar of future gynecologic oncology [108].

### 6.11. Emerging Advanced AI Architectures

Beyond current ML and DL approaches, several advanced AI methodologies are beginning to influence cancer research and hold promise for gynecological oncology. Vision transformers (ViTs) are increasingly applied in histopathology to capture long-range spatial dependencies in tissue images, outperforming traditional CNNs in some contexts. Self-supervised learning (SSL) offers powerful strategies in low-data medical environments by pre-training on unlabeled datasets before fine-tuning on smaller labeled cohorts, which is particularly relevant for rare gynecologic cancer subtypes. Graph neural networks (GNNs) enable the modeling of complex patient–gene–phenotype networks, making them attractive for precision oncology and biomarker discovery. Incorporating these emerging methods into gynecological cancer research could accelerate progress toward more robust, generalizable, and clinically relevant AI solutions.

## 7. Conclusions

The burden due to gynecological cancer (i.e., breast, ovarian, and cervical cancer) still remains a major challenge to the health outcomes of females, especially in regions that lack early inspection and progressive care [109]. The time of detection and the variability of treatment outcomes require the development of innovative solutions capable of enhancing the accuracy of diagnoses and the tailoring of treatment regimens [110]. ML is turning out to be a game-changer in this setting. We have discussed how ML approaches, ranging from decision trees and SVMs to DL networks, are being applied across gynecologic cancers to improve clinical detection, outcome forecasting, clinical risk stratification, and disease monitoring. ML is helpful in analyzing mammograms and genetic subtypes in BC, and ML is enhancing HPV testing and Pap smear examination in cases of cervical cancer. In regard to OC, ML is beginning to assist in early diagnosis, identifying secretive features of biomarkers and scanning them radio-diagnostically [111].

There are challenges associated with the concept of ML, namely data quality, model transparency, and real-world validation. However, things are bright ahead. Through the development of XAI, FL, and multi-omics integration, the field of gynecologic cancer treatment may experience a transformation that will help in detecting cancer earlier, triaging patients more accurately, and making truly personalized decisions regarding treatment [112]. In the future, it will be best to work in collaboration with clinicians, data scientists, ethicists, and policy makers; only together can we ensure that ML technologies will be immensely strong, safe, ethical, and inclusive. Step by step, we can ensure that the concern of cancer care remains timely and precise, no matter who you are or where you live [113].

The integration of ML into gynecologic oncology has the potential to transform routine practice. Beyond enhancing diagnostic precision, ML models can refine risk stratification, predict treatment outcomes, and personalize care pathways for patients with breast, ovarian, and cervical cancers [114]. For gynecologic oncologists, the integration of ML into daily practice offers practical advantages—earlier detection of OC through biomarker integration, fertility-sparing treatment planning in young BC and cervical cancer patients, and risk-adapted surveillance for recurrent disease. By bridging computational advances with clinical workflows, ML holds the potential to deliver more individualized, equitable, and patient-centered gynecologic cancer care.

## Figures and Tables

**Table 1:** Major ML approaches and applications in gynecological oncology.

ML Approach	Key Algorithms/Models	Core Features	Applications in Gynecologic Oncology
Supervised Learning	Logistic Regression, Decision Trees, Random Forests, Support Vector Machines (SVMs), k-Nearest Neighbors (k-NN)	Trains on labeled datasets (input → known output)	Predicting recurrence risk in BC using gene expression data Stratifying OC patients based on CA-125 levels and imaging Automating Pap smear classification for cervical cancer
Unsupervised Learning	k-Means Clustering, Hierarchical Clustering, Principal Component Analysis (PCA)	Finds hidden patterns in unlabeled data	Discovering novel molecular subtypes of ovarian/endometrial cancer Clustering cervical cancer patients by HPV genotypes Identifying treatment response subgroups in endometrial cancer
Deep Learning (DL)	Convolutional Neural Networks (CNNs), Recurrent Neural Networks (RNNs), Autoencoders	Multi-layer neural networks that learn features automatically	CNNs for automated detection of breast tumors in mammograms Pap smear and colposcopy image classification for cervical cancer Integrating genomics + histopathology in OC outcome prediction
Hybrid/Ensemble Models	Gradient Boosting Machines (XGBoost, Light GBM), Ensemble DL models	Combine multiple algorithms to improve accuracy and reduce bias	Multi-omics integration for OC prognosis Risk stratification tools combining imaging + EHR data Prognosis modeling across multiple gynecologic cancers

**Table 2:** Overview of ML algorithms applied in gynecological cancers: application areas, data sources, clinical impact, and supporting evidence.

ML Algorithm	Application Area	Gynecologic Cancer Type	Data Source	Clinical Impact	References
Decision Trees	Risk classification, interpretability	Cervical, Endometrial	Clinical records, HPV data	Transparent decision rules for triage and histological subtyping	[20,21,46]
Random Forest	Survival prediction, subtype classification	Breast, Ovarian	Genomic and histopathology data	Robust ensemble learning; improved prognostic modelling	[31,32,36,46]
Support Vector Machine (SVM)	Lesion detection, subtype prediction	Breast, Cervical, Ovarian	Imaging, gene expression, biomarkers	High accuracy in high-dimensional, small-sample data	[24,25,29]
Convolutional Neural Networks (CNN)	Image-based diagnostics	Cervical, Breast, Endometrial	Mammograms, Pap smears, MRIs	Automated, accurate image classification for early diagnosis	[47,48,49]
LASSO Regression		Ovarian	Proteomics, miRNAs	Reduces overfitting while enhancing marker-based prediction	[50,51,52]
Recurrent Neural Networks (RNN)	Sequence-based analysis	Ovarian	Gene expression time series	Models longitudinal or time-varying clinical data	[45,53,54]
PCA/K-Means (Unsupervised)	Tumor subtyping, pattern discovery	Endometrial, Ovarian	Multi-omics, expression clustering	Discovers hidden patterns and new cancer subgroups	[38,39,55]
XGBoost	Risk stratification, biomarker evaluation	Cervical, Ovarian	Combined omics and clinical data	High performance with imbalanced datasets	[31,56]

**Table 3:** Clinical applications of ML across gynecological cancers: data modalities, algorithmic strategies, and translational outcomes.

Cancer Type	Application	Data Type	ML Techniques	Clinical Impact	References
Breast	Tumor detection	Mammography, MRI	CNN, SVM	Early, accurate diagnosis	[18,45,62]
	Recurrence prediction	Gene expression	Random Forest, ANN	Personalized treatment planning	[32,61,72]
Cervical	HPV-based risk prediction	HPV genotyping, clinical records	Logistic Regression, SVM	CIN progression risk stratification	[22,33,56]
	Pap smear analysis	Cytology images	CNN, U-Net	Automated screening, consistency	[27,71]
Ovarian	Prognosis, biomarkers	Proteomics, miRNA	SVM, XGBoost	Improved early-stage detection	[28,30,41]
	Tumor classification, prognosis	MRI, CT, genomics	Radio-genomics, Random Survival Forests	Treatment response prediction	[52,54]
Endometrial	Subtype classification, survival	Histopathology, gene expression	CNN, PCA	Accurate risk group identification	[38,41]
	Tumor heterogeneity and biomarker discovery	Multi-omics and clustering	K-Means, Hierarchical Clustering	Insights into novel molecular subgroups	[39,76]

**Table 4:** Comparative overview of ML tools in gynecological cancer care: from research to clinical.

ML Approach	Research Setting Use Case	Clinical Setting Example	Validation Status	Advantages	Limitations/Barriers
CNN (Deep Learning)	Automated Pap smear classification	Cervical image analysis in low-resource clinics	Retrospective + pilot clinical	High accuracy in image tasks	Requires large labeled datasets
Random Forest	Ovarian cancer risk prediction from omics data	Predicting recurrence from histology	Retrospective validation	Robust to noise, handles missing data	Interpretability lower than decision trees
XGBoost	CA-125 + miRNA-based early detection	Decision support for screening protocols	Research-phase	Handles imbalanced data well	Needs careful tuning; overfitting risk
SVM	Gene expression-based subtype classification	MRI-based tumor segmentation	Preclinical	Good in high-dimensional settings	Not scalable to very large datasets
LASSO Regression	miRNA signature selection	Prognostic modeling in ovarian cancer	Retrospective cohort studies	Simplicity; feature reduction	May underperform in nonlinear problems
Radiomics + ML Fusion	Texture-based lesion characterization from imaging	BRCA status prediction from MRI/CT	Early-phase clinical trials	Links imaging to genomics (radio genomics)	Data harmonization between centers is challenging
Unsupervised Learning	Identifying novel subtypes from multi-omics datasets	Tumor classification beyond histology	Research exploration	Discovers hidden patterns without prior labels	Interpretation and reproducibility

**Table 5:** Evaluation of metrics and external validation status in gynecologic oncology ML/DL studies.

Model Type	Application	Metrics Reported	External Validation
Decision Trees	BC risk stratification	Accuracy, Sensitivity	No
SVM	Cervical cytology classification	AUC, Specificity	Single-center only
Random Forests	OC biomarker prediction	F1-score, Calibration	No
CNN (DL)	Pap smear image analysis	AUC, Sensitivity, Specificity	Rarely multi-center
Transformer (DL)	Histopathology subtype classification	AUC, Precision	Early pilot only

## Abbreviations

AI: Artificial Intelligence; ANN: Artificial Neural Network; BC: Breast Cancer; BRCA1/2: Breast Cancer Gene 1 and 2; CDSS: Clinical Decision Support Systems; CIN2+: Cervical Intraepithelial Neoplasia Grade 2 or Higher; CNN: Convolutional Neural Network; DDSM: Digital Database for Screening Mammography; DL: Deep Learning; HER: Electronic Health Record; EMA: European Medicines Agency; ER/PR: Estrogen Receptor/Progesterone Receptor; FL: Federated Learning; GDPR: General Data Protection Regulation; HER2: Human Epidermal Growth Factor Receptor 2; HIPAA: Health Insurance Portability and Accountability Act; HPV: Human Papillomavirus; LASSO: Least Absolute Shrinkage and Selection Operator; ML: Machine Learning; OC: Ovarian Cancer; PCA: Principal Component Analysis; RNN: Recurrent Neural Network; RSF: Random Survival Forest; SHAP: Shapley Additive Explanations; SVM: Support Vector Machine; TCGA: The Cancer Genome Atlas; ViTs: Vision Transformers; XAI: Explainable Artificial Intelligence; XGBoost: Xtreme Gradient Boosting.
